# Benchmarks after bowel resection in Crohn’s disease: implementing clinically meaningful health-related quality of life targets

**DOI:** 10.1007/s00464-025-11983-z

**Published:** 2025-07-23

**Authors:** Thomas E. Ueland, Megan M. Shroder, Samuel A. Younan, Sara N. Horst, Allison B. McCoy, Justin M. Bachmann, Alexander T. Hawkins

**Affiliations:** 1 Vanderbilt University School of Medicine, Nashville, TN USA; 2 Division of General Surgery, Vanderbilt University Medical Center, Nashville, TN USA; 3 Department of Gastroenterology, Hepatology, and Nutrition, Vanderbilt University Medical Center, Nashville, TN USA; 4 Department of Biomedical Informatics, Vanderbilt University Medical Center, Nashville, TN USA; 5 Department of Medicine, Division of Cardiovascular Medicine, Vanderbilt University Medical Center, Nashville, TN USA; 6 Division of General Surgery, Section of Colon & Rectal Surgery, Vanderbilt University Medical Center, Nashville, TN USA

**Keywords:** Patient-reported outcome measures, Crohn’s disease, PASS, MCID

## Abstract

**Background:**

Patient-reported outcome measure scores must be interpretable to be effective in a clinical setting. In this retrospective cohort study, we sought to establish the minimum clinically important difference (MCID) and patient acceptable symptom state (PASS) for health-related quality of life in Crohn’s disease and to apply these thresholds to patients undergoing Crohn’s-related bowel resection.

**Methods:**

Eligible participants were adults with Crohn’s disease completing a patient-reported outcome measure and an additional anchor question about digestive satisfaction or change from a prior clinical visit. Multiple questionnaires were evaluated for alignment with digestive satisfaction, and the best-performing short inflammatory bowel disease questionnaire (sIBDQ) was retained for further analysis. Clinically meaningful threshold ranges were established for the sIBDQ through anchor- and distribution-based methodologies. These thresholds were applied to patients undergoing Crohn’s-related bowel resection in three areas: rates of target score achievement at postoperative time points, factors associated with sIBDQ scores at final follow-up, and accessibility in the electronic medical record.

**Results:**

There were 492 and 228 responses included in the PASS and MCID analyses, respectively. For the sIBDQ, the PASS ranged from 51 to 63, while the MCID ranged from 6.5 to 9.5. In the 215 patients undergoing Crohn’s-related bowel resection, the preoperative sIBDQ median (IQR) was 49 (30, 69) compared to postoperative scores of 56 (40, 73) at 1–12 months, 56 (39, 73) at 13–24 months, and 58 (42, 73) at more than 24 months. The PASS was achieved in 179 (84%) patients for at least one postoperative time point. Preoperative sIBDQ score and male sex were associated with higher quality of life at final follow-up.

**Conclusion:**

In Crohn’s disease, clinically meaningful targets for quality of life may complement traditional metrics when monitoring progress after operative intervention.

**Graphical abstract:**

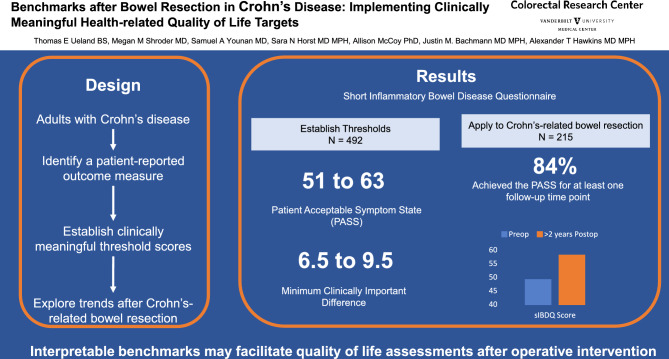

**Supplementary Information:**

The online version contains supplementary material available at 10.1007/s00464-025-11983-z.

Health-related quality of life is an important therapeutic target in Crohn’s disease [1–3]. Because bowel resection is not curative and up to 35% of patients require multiple resections within 10 years, longitudinal monitoring of disease burden in relation to prior states is essential [4]. Patient-reported outcome measures (PROMs) quantify health-related quality of life as experienced directly by patients, complementing traditional disease activity markers through insight into individualized impact on daily functioning [5]. A wide range of disease-specific and generic PROM instruments have been used to monitor progress after clinical interventions over time [6].

Adoption of PROMs requires approaches for characterizing meaningful achievement. Variation in scoring scales, disease burden, and the expected benefit of interventions ensures that no universal numeric target exists for PROMs. Further, statistical significance does not always translate to noticeable value for patients [7]. One strategy for improving the interpretation of numeric PROM scores is through clinically meaningful outcome thresholds, such as the Patient Acceptable Symptom State (PASS) for single score values and the Minimum Clinically Important Difference (MCID) for change scores. The Patient Acceptable Symptom State (PASS) represents a score where patients are satisfied with their disease status [8, 9] and has been framed as a benchmark for success with operative or pharmacologic interventions [10–12]. The MCID represents the smallest change in score over time that is meaningful to patients [13].

This study aimed to establish clinically meaningful outcome thresholds for health-related quality of life in Crohn’s disease and to better understand score trends over time after Crohn’s-related bowel resection. We hypothesized that meaningful health-related quality of life targets would be achieved after bowel resection.

## Materials and methods

### Study design and population

**Fig. 1:**
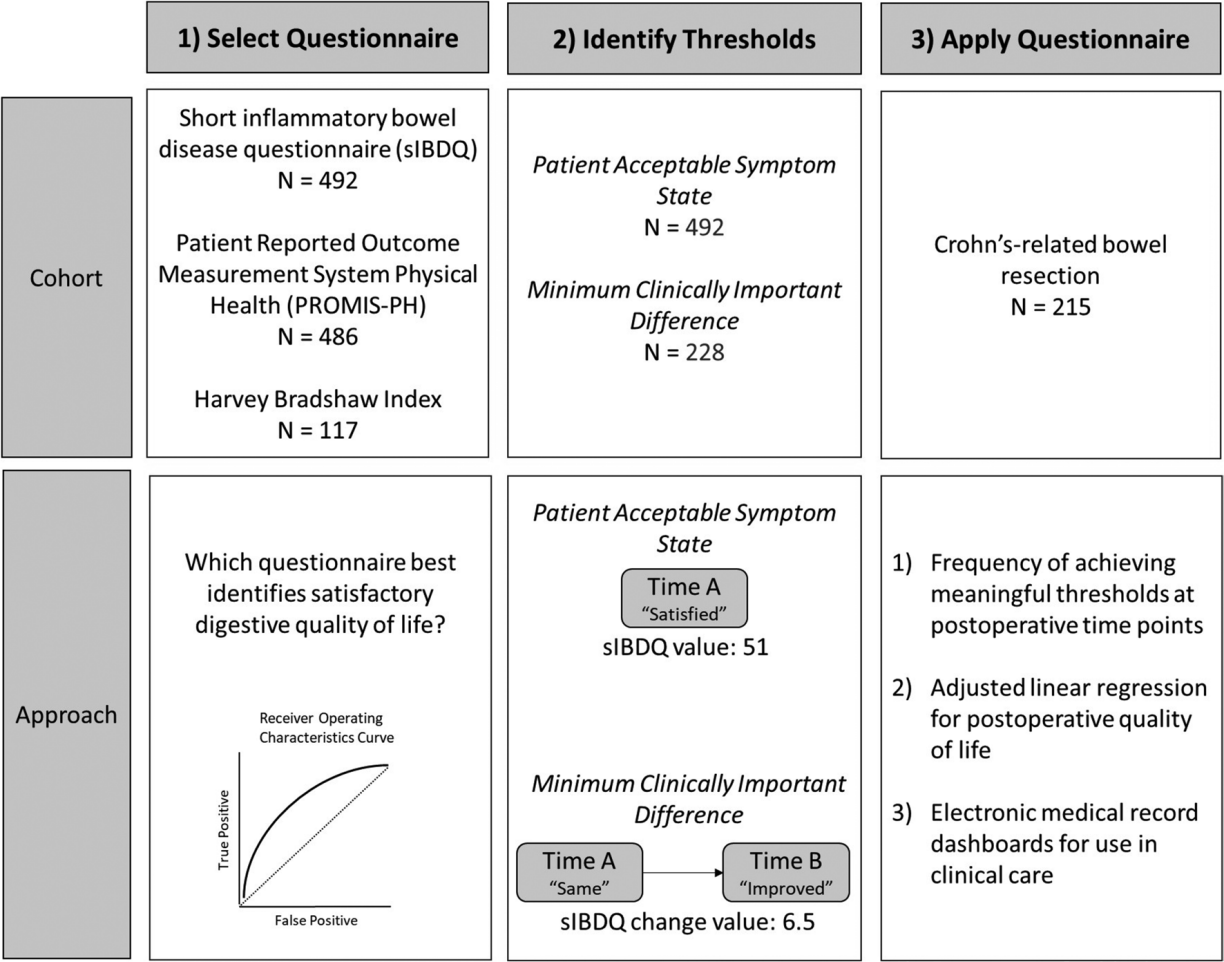
Overview of study design

This retrospective cohort study first evaluated three candidate questionnaires for congruence with a self-reported satisfactory disease state. For the best-performing questionnaire, we established clinically meaningful thresholds (MCID and PASS) using anchor- and distribution-based methodologies [11, 14, 15]. Finally, we quantified rates of achieving these thresholds among Crohn’s-related bowel resection patients and fit multivariable regression models for quality of life scores at final follow-up. Trend graphs were integrated into electronic medical record dashboards to facilitate accessibility for future patients. The complete study design is summarized in Fig. 1.

The study population consisted of adult patients with Crohn’s disease (International Classification of Diseases, 10th revision, Clinical Modification code K50) who completed a clinical encounter at our tertiary care center between 2020 and 2024. At our institution, PROMs are routinely assigned to patients based on their encounter location and associated diagnosis codes. The questionnaires are typically completed through the electronic patient portal on a personal device before the encounter date but can also be completed in the clinic waiting room [16]. Patients with diverticulitis, colon cancer, rectal cancer, or anal cancer during the study period were excluded since coexisting gastrointestinal diagnoses may also influence digestive quality of life. Patients assigned diagnosis codes for Ulcerative Colitis or indeterminate colitis in addition to Crohn’s disease were excluded to minimize misclassification bias. This study was approved by the Vanderbilt University Medical Center Institutional Review Board (IRB#231872) with a license agreement for the sIBDQ (MILO000443).

### Questionnaire selection

Three questionnaires were compared in this study: the Short Inflammatory Bowel Disease Questionnaire (sIBDQ), the Patient-Reported Outcomes Measurement System Global 10 Physical Health T-score (PROMIS-PH), and the Harvey Bradshaw Index. The sIBDQ is a disease-specific instrument including ten items that best explain the variance in the original 36-item Inflammatory Bowel Disease Questionnaire [17, 18]. Scores range from 10 (poor) to 70 (optimal) with domains of bowel symptoms, systemic symptoms, emotional functioning, and social functioning. The PROMIS-PH is a summary score generated from the PROMIS-10 items that address physical function, fatigue, and pain [19, 20]. It is standardized to a T-score such that the mean ± standard deviation T-score of the United States population is 50 ± 10. While not specific to digestive symptoms, the PROMIS-PH is generalizable across many diseases and has been applied previously in Crohn’s disease [21, 22]. The Harvey Bradshaw Index approximates disease severity through symptoms, well-being, and complications [23].

The criterion used for the questionnaire selection in this study was area under the receiver operating characteristics curve (AUC). For each candidate questionnaire, a logistic regression model was fit with the questionnaire score as a covariate and an outcome of responding “Yes” to a self-reported satisfaction question completed within 14 days (“Today, are you satisfied with your digestive quality of life”). Among all candidate questionnaires, the sIBDQ demonstrated the largest AUC and was included in the remainder of the analyses.

### Clinically meaningful outcome thresholds

Eligibility for the PASS analysis required completion of an sIBDQ and the satisfaction anchor question within 14 days. An anchor-based method interprets the PROM score in the context of an external question that is answered at the same time. By capturing more intuitive health states, this external question helps to “anchor” the interpretation of the PROM score. In this study, the external anchor question for the PASS threshold was “Today, are you satisfied with your digestive quality of life?” and the external anchor question for the MCID threshold was “Since your last visit, do you feel that your digestive health has improved, stayed the same, or worsened?”. If a patient responded “Yes” to the satisfaction anchor question at one time point and “No” at a different time point, both entries were included. For patients with multiple sIBDQ scores associated with the same satisfaction anchor response, the median value was taken. Eligibility for the MCID analysis required completion of the sIBDQ and the change anchor question at a visit, while also completing an sIBDQ at their previous visit between 3 and 15 months prior to the inclusion visit. We limited our MCID analysis to those with substantial disease impact at baseline, as meaningful improvement is unlikely to be observed among those with minimal disease impact initially [11]. Thus, only patients who reported an sIBDQ score below the PASS at their baseline visit were included for the MCID analysis.

The PASS and MCID were derived through both anchor- and distribution-based methods. The anchor-based strategies identified an sIBDQ score that maximized sensitivity and specificity when discriminating between responses to the anchor question (“Yes” vs “No” for the PASS; “Improved” vs “Stayed the same” for the MCID). For the PASS, distribution-based strategies identified the 75th percentile among patients who reported a satisfactory disease state. For the MCID, distribution-based strategies included the average change among those who improved and the change difference between those who improved versus those who stayed the same.

### Application to Crohn’s-related bowel resection patients

The Crohn’s-related bowel resection analysis included adult patients with Crohn’s disease who underwent bowel resection during the study period in addition to an sIBDQ completed in the postoperative period (Supplementary Material). Postoperative time periods were binned into 1–12 months, 13–24 months, and more than 24 months. For patients who reported multiple scores during those time points, the median of the scores was taken. An eligible preoperative entry occurred within 90 days prior to the procedure, with the closest encounter to the date of procedure taken if multiple were available.

Next, we fit a multivariable linear least squares regression model with an outcome of sIBDQ score at final follow-up. Covariates were age, sex, ever tobacco use, body mass index (BMI), disease duration, disease pattern, disease location, resection history, operative approach, immunosuppressing medication status, preoperative sIBDQ score, questionnaire completion method at final follow-up, and the presence of an ostomy at final follow-up. All covariates except for ostomy presence and questionnaire completion method (in clinic versus electronic portal) were obtained from the preoperative visit. Disease phenotype information was documented by providers during the visit via a standardized form in the electronic medical record, while operative approach was categorized as open or laparoscopic based on the CPT4 code. Immunosuppressing medication status was defined as an active prescription for an immunomodulator, biologic, or corticosteroid within 30 days of the encounter (Supplementary Material). In a sensitivity analysis, we examined sIBDQ scores at final follow-up when stratified by postoperative endoscopic disease recurrence and postoperative 30-day all-cause readmission status.

To facilitate the use of PROM scores in practice, we integrated dashboards in the electronic medical record depicting trends in quality of life over time. Visualizations aimed to show both within-patient scores through follow-up and aggregate scores across all patients undergoing Crohn’s-related bowel resection.

## Results

### Clinically meaningful outcome thresholds

**Fig. 2:**
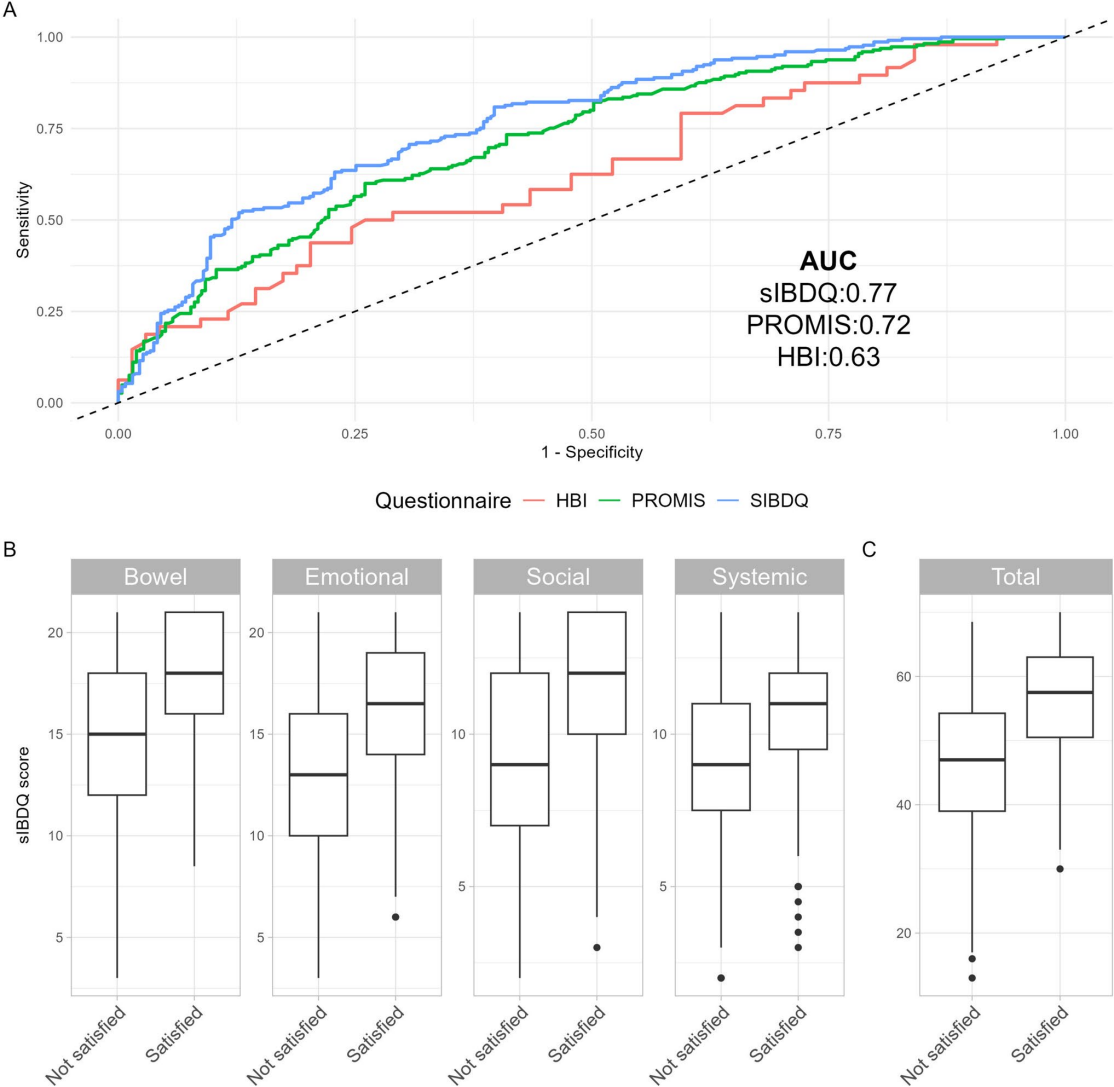
Questionnaire selection for clinically meaningful thresholds. **A** Receiver Operating Characteristics curve for candidate questionnaires relative to responding “Yes” to the satisfaction anchor question. **B** Box plot of sIBDQ subscale and total scores stratified by response to the satisfaction anchor question. *sIBDQ* short inflammatory bowel disease questionnaire. *PROMIS* patient-reported outcomes measurement information system global 10 physical health T-score. *HBI* Harvey Bradshaw index. *AUC* area under the receiver operating characteristics curve

**Fig. 3:**
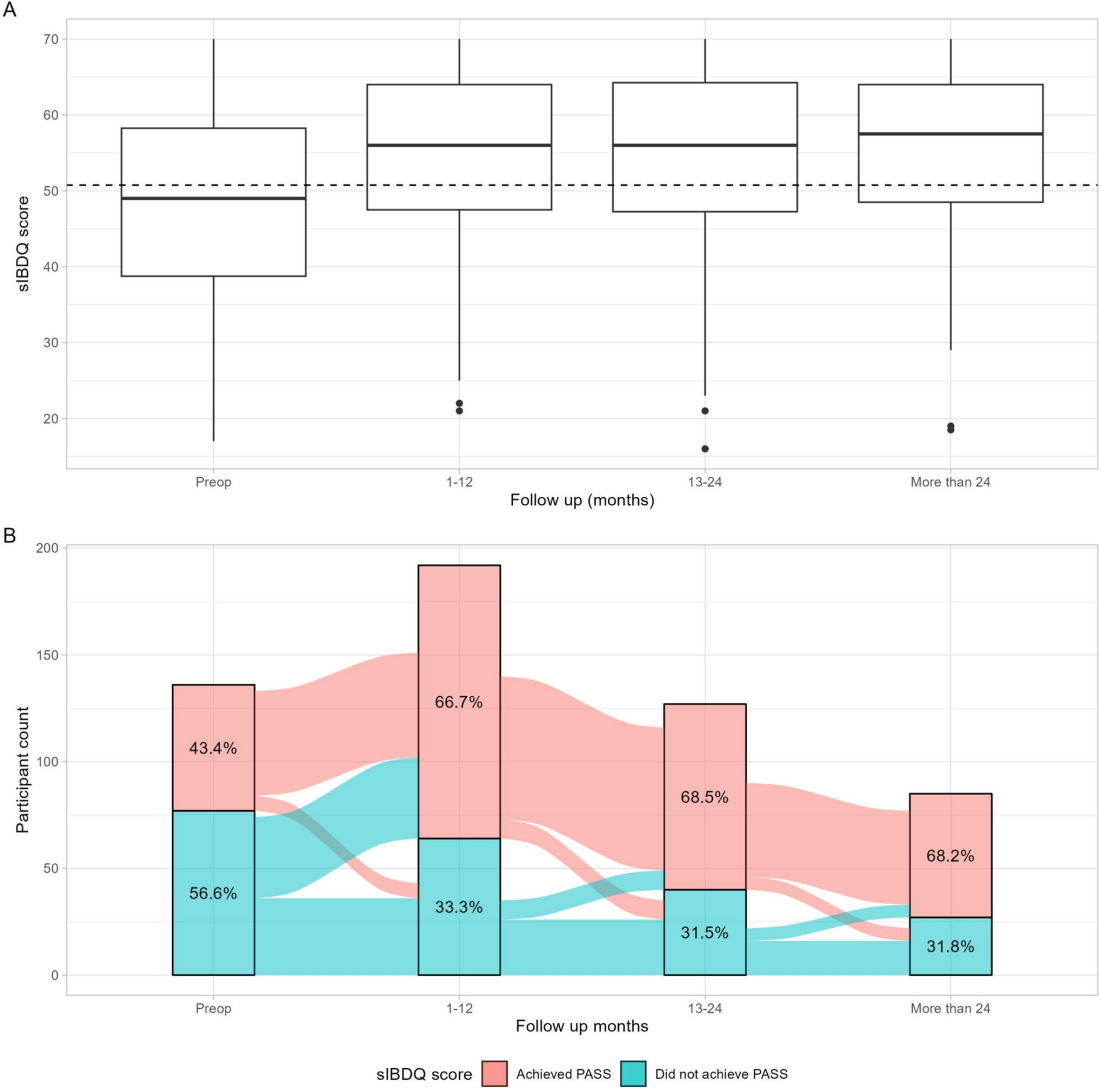
sIBDQ scores throughout postoperative follow-up time points. **A** Boxplot of sIBDQ scores. Horizontal dashed line represents score corresponding to Patient Acceptable Symptom State (51). **B** Alluvia plot of sIBDQ scores. *Preop* baseline preoperative visit. *sIBDQ* short inflammatory bowel disease questionnaire. *PASS* patient acceptable symptom state

The sIBDQ was retained for calculation of clinically meaningful thresholds as it maximized discriminatory performance for a satisfactory disease state (AUC 0.77) relative to the PROMIS-PH (AUC 0.72) and the Harvey Bradshaw Index (AUC 0.63) (Fig. 1). There were 225 to 492 sIBDQ responses included in the PASS calculations and 115 to 228 for the MCID calculations, depending on the methodology used (see Figs. 2, 3).

Of those completing a satisfaction anchor, a minority of patients reported satisfaction with their digestive quality of life (225 (45.7%), Table 1). Satisfied patients were less likely to have concurrent corticosteroid use (36 (16%) vs. 80 (30%), *p* < 0.01), but no differences in resection history or disease patterns were observed. Median sIBDQ scores were higher for patients who were satisfied relative to those who were dissatisfied for the total score (58 vs. 47) as well as all subscales: Bowel (18 vs. 15), Systemic Symptoms (11 vs. 9), Social (12 vs. 9), and Emotional (17 vs. 13) (Fig. 1). The PASS values were 51 by anchor and 63 by distribution methods (Table 2).

**Table 1:** Clinical characteristics across satisfaction responses

Characteristic	Overall *N* = 492	Not satisfied *N* = 267	Satisfied *N* = 225	*p*-value
Age (years)	41 (31, 54)	42 (31, 55)	40 (30, 51)	0.13
Sex				0.70
Female	258 (52%)	143 (54%)	115 (51%)	
Male	234 (48%)	124 (46%)	110 (49%)	
BMI (kg/m^2^)	26 (22, 30)	26 (22, 30)	26 (22, 30)	> 0.90
Insurance				0.50
Private	329 (67%)	173 (65%)	156 (70%)	
Government	122 (25%)	72 (27%)	50 (22%)	
Other	39 (8.0%)	22 (8.2%)	17 (7.6%)	
Tobacco use				0.50
Current	103 (21%)	61 (23%)	42 (19%)	
Prior	109 (23%)	59 (22%)	50 (23%)	
Never	271 (56%)	143 (54%)	128 (58%)	
Concurrent steroids	116 (24%)	80 (30%)	36 (16%)	< 0.01
Concurrent immunomodulators or biologics	211 (43%)	114 (43%)	97 (43%)	> 0.90
sIBDQ score	52 (44, 59)	47 (39, 55)	58 (51, 63)	< 0.01
Extraintestinal manifestations	32 (6.5%)	15 (5.6%)	17 (7.6%)	0.50
Pattern				0.70
Inflammatory	108 (23%)	60 (24%)	48 (22%)	
Penetrating and/or stricturing	365 (77%)	195 (76%)	170 (78%)	
Location				0.80
Colonic	103 (22%)	52 (21%)	51 (24%)	
Ileal	147 (31%)	81 (32%)	66 (31%)	
Ileocolonic	217 (46%)	118 (47%)	99 (46%)	
Resection history				0.80
No prior resection	298 (61%)	160 (60%)	138 (61%)	
Prior resection(s)	194 (39%)	107 (40%)	87 (39%)	

Values represent median (IQR); *n* (%). *p* values represent Wilcoxon rank sum test; Fisher’s exact test. *BMI* body mass index. *HBI* Harvey Bradshaw index. *sIBDQ* short inflammatory bowel disease questionnaire. *PASS* patient acceptable symptom state

**Table 2:** Clinically meaningful thresholds for the short inflammatory bowel disease questionnaire

Threshold	Methodology	Description	Questionnaire count	Score value
Patient acceptable symptom state (PASS)	75th percentile	75th percentile sIBDQ score among those who responded “Yes” to the PASS anchor question	225	63
	ROC curve	sIBDQ score with largest sensitivity and specificity when discriminating between those who responded “Yes” from those who responded “No” to the PASS anchor question	492	51
Minimum clinically important difference (MCID)	ROC curve	sIBDQ score with largest sensitivity and specificity when discriminating between those who responded “Improved” from those who responded “Stayed the same” to the MCID anchor question	228	6.5
	Average change	Mean sIBDQ change score among patients who responded “Improved” to the MCID anchor question	115	9.5
	Change difference	Difference in mean sIBDQ change scores between patients who responded “Improved” and patients who responded “Stayed the same” to the MCID anchor question	228	8.0

*ROC* receiver operating characteristics. sIBDQ short inflammatory bowel disease questionnaire

PASS anchor question: “Today, are you satisfied with your digestive quality of life?”

MCID anchor question: “Since your last visit, do you feel that your digestive health has improved, stayed the same, or worsened?”

In the MCID analysis, the difference in time between the baseline visit and inclusion visit was a median (IQR) of 3.9 (2.1, 5.7) months. Relative to their previous visit, there were 115 (50.4%) patients who improved and 113 (49.6%) patients who stayed the same. The MCID threshold values were 6.5 by anchor methods and ranged from 7.96 to 9.52 by distribution methods (Table 2).

### Application to Crohn’s-related bowel resection

After threshold derivation, we applied the PASS to patients undergoing Crohn’s-related bowel resection. Given observed variability between the anchor- and distribution-based values, we preferred the anchor-based value consistent with previous recommendations [14].

There were 215 patients included in the Crohn’s-related bowel resection analysis with an sIBDQ completed at median (IQR) follow-up of 20.9 (6.6, 32.1) months after the operation (Supplementary Material). At final follow-up, 76 (35.3%) of patients had an ostomy present. Compared to a preoperative sIBDQ median (IQR) of 49 (30, 69), the postoperative scores were 56 (40, 73) at 1–12 months, 56 (39, 73) at 13–24 months, and 58 (42, 73) at more than 24 months (Fig. 1). When considering all follow-up time points, there were 179 (84%) patients who achieved the PASS for at least one time point. There was substantial variability among patients reporting multiple scores for a time point, as the within-patient standard deviation was greater than the MCID in 17% (13–24 month) to 33% (1–12 month) of patients (Supplementary Material). In the multivariable regression analysis, the preoperative sIBDQ (β coefficient [95% CI], 0.51 [0.38–0.64]) and male sex (β coefficient [95% CI], 3.0 [0.36–5.7]) were associated with higher postoperative sIBDQ scores (Table 3). Patients with any endoscopic evidence of recurrence during follow-up reported median (IQR) sIBDQ scores of 57 (43, 70) at final follow-up, while those with a 30-day all-cause readmission reported median (IQR) sIBDQ scores of 55 (42, 68) (Supplementary Material).

**Table 3:** Linear regression model for short inflammatory bowel disease questionnaire scores at final follow-up

Variable	Beta [95% CI]	*p*-value
Age (years)	0.01 (− 0.09 to 0.12)	0.83
Sex: male (reference: female)	3.0 (0.36 to 5.7)	0.03
Ever tobacco use	− 2.5 (− 5.3 to 0.31)	0.08
BMI (kg/m2)	− 0.20 (− 0.42 to 0.03)	0.09
sIBDQ completed via electronic portal (reference: in clinic)	4.6 (− 0.57 to 9.7)	0.08
Disease duration (years)	0.07 (− 0.07 to 0.21)	0.31
Study resection: open approach (reference: laparoscopic)	1.1 (− 1.8 to 4.0)	0.46
Concurrent immunomodulators or biologics or steroids	0.30 (− 2.8 to 3.4)	0.85
Disease pattern: penetrating and/or stricturing (reference: inflammatory)	− 1.9 (− 5.8 to 1.9)	0.33
Disease location (reference: colonic)		
Ileal	− 1.8 (− 6.2 to 2.6)	0.42
Ileocolonic	− 1.9 (− 6.1 to 2.4)	0.39
Preop sIBDQ score	0.51 (0.38 to 0.64)	< 0.01
Ostomy present at final follow-up	0.90 (− 2.1 to 3.9)	0.56
Resection history	− 2.1 (− 5.0 to 0.83)	0.16

*sIBDQ* short inflammatory bowel disease questionnaire. *BMI* body mass index

**Fig. 4:**
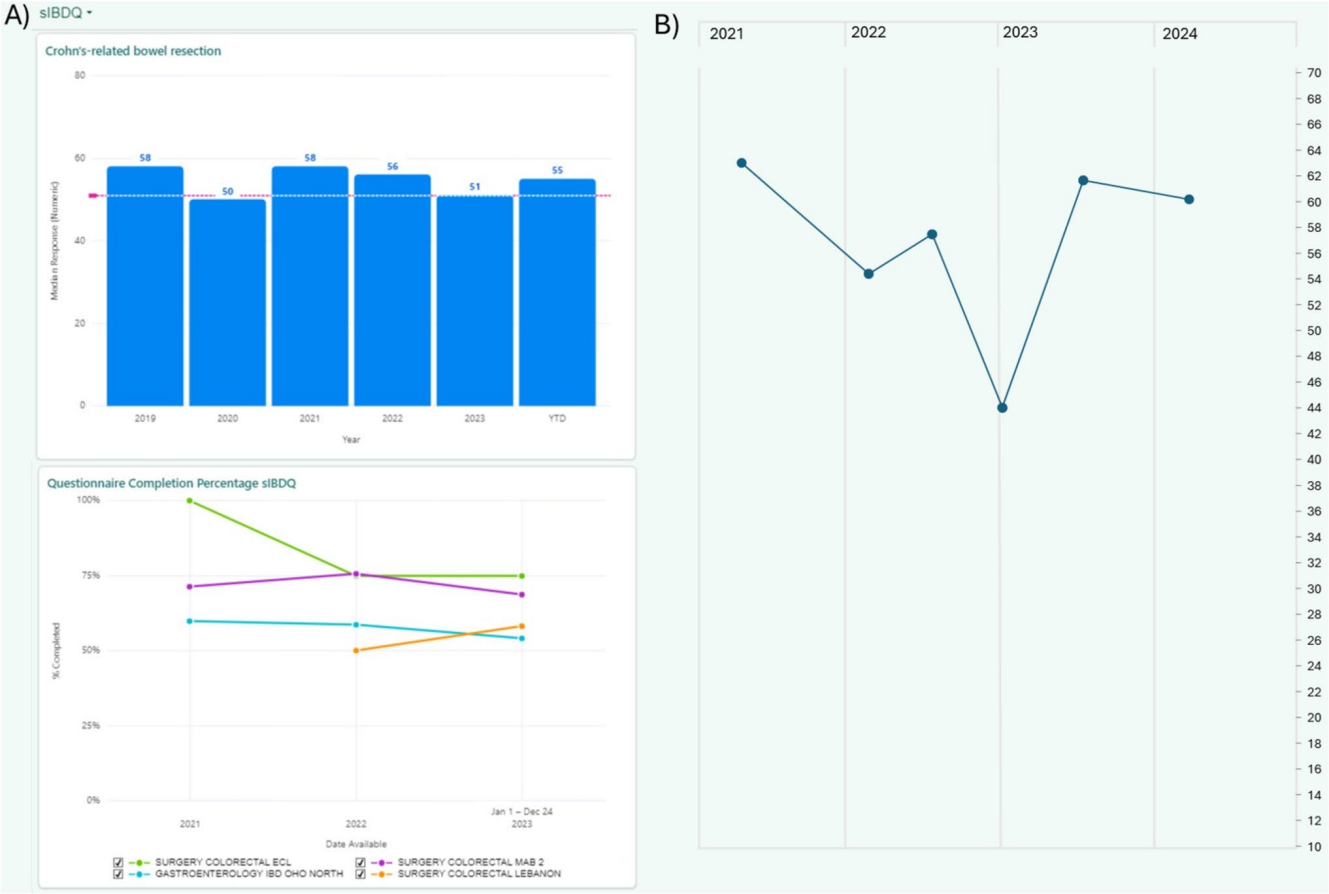
Electronic medical record visualizations for health-related quality of life in Crohn’s disease. **A** Visualization of median sIBDQ scores among all patients undergoing Crohn’s-related bowel resection (top panel) and questionnaire completion rate by clinic site (bottom panel). Dashed line represents Patient Acceptable Symptom State for the sIBDQ. **B** Score trends of a sample patient’s sIBDQ scores over time available within a patient’s chart. *sIBDQ* Short Inflammatory Bowel Disease Questionnaire

The electronic medical record integration depicts multiple perspectives for PROM scores (Fig. 4). Dashboard views are customizable by the user to focus on specific clinical encounter sites, patient characteristics, and provider patient panels. At the within-patient level, graphs that show score changes over time are available for review with patients during the encounter, and values can be pulled into clinical documentation notes for the visit.

## Discussion

This study derived health-related quality of life targets for Crohn’s disease using a patient-reported outcome measure, the sIBDQ. Most patients undergoing Crohn’s-related bowel resection reported scores above the Patient Acceptable Symptom State in the postoperative period, suggesting that satisfactory quality of life is often achieved after operative intervention. Preoperative sIBDQ scores were associated with scores at final follow-up; while indicating a possible role for baseline quality of life when discussing expectations about postoperative trajectories, external validation is needed. Trend graphs integrated into the electronic medical record facilitate accessibility during clinical encounters. Overall, this study provides a framework for adopting quality of life assessments after Crohn’s-related bowel resection and applies this framework for target scores grounded in satisfaction (PASS) or meaningful change (MCID).

The importance of health-related quality of life for longitudinal monitoring in Crohn’s disease has been reflected in international guideline statements [2], prolific growth of disease-specific PROMs [24], and primary endpoints in randomized controlled trials [25, 26]. Factors related to both questionnaire performance and practical barriers must be considered when adopting quality of life questionnaires in a clinical setting. While disease-specific PROMs like the sIBDQ capture constructs that are tailored to a specific group, lack of applicability across diseases means that multiple questionnaires must be used for distinct patient populations. Generic PROMs like the PROMIS-PH can apply broadly, but they may miss components that are particularly important only for some diseases. We showed that the sIBDQ outperformed both the PROMIS-PH and the Harvey Bradshaw Index for identifying a satisfactory disease state in Crohn’s disease. However, an AUC in the acceptable range suggests that constructs relevant to satisfaction were missing or differentially weighted across participants. This may improve by increasing the number of questionnaire items, but at the expense of an additional time burden on patients. Providers or researchers must critically evaluate questionnaire performance in the context of survey fatigue, generalizability to other patient populations, and ease of administration [27, 28]. While our study supports disease-specific quality of life as a better indicator of digestive satisfaction in Crohn’s disease relative to generic instruments, there are other similar questionnaires available and no consensus about the best one to use [29, 30].

By linking numeric scores with interpretable health states, the MCID and PASS provide boundaries for achievement with PROMs much like scoring systems facilitate the grading of endoscopic or laboratory severity. Our study offers targets using both single score sIBDQ values (PASS, range 51 to 63) and change score values (MCID, range 6.5 to 9.5). In the initial sIBDQ validation study, mean scores of patients with active Crohn’s disease (range 40–49) were lower than those with inactive disease (range 47 to 58) [18]. Subsequently, Siffledeen et al. [31] reported a mean sIBDQ score of 58.2 among Crohn’s disease patients with optimal control as well as a difference in means of 15.2 relative to patients with suboptimal control. Importantly, they found variation between strategies for identifying disease control; complete agreement between patient perceptions, clinical perceptions, and objective red flags was achieved in only 15.6% of Crohn’s disease patients. Our study anchored sIBDQ scores to self-perceived satisfactory states instead of laboratory or endoscopic remission. Patients may report satisfactory quality of life without achieving endoscopic healing. Conversely, normalization of inflammatory markers does not guarantee the absence of disease burden on daily functioning. In light of recommendations supporting a multi-faceted approach to monitoring after interventions in Crohn’s disease [2, 3], we believe that quality of life assessments can complement physician-centric metrics when seeking a shared understanding of disease impact.

We envision two practical applications of interpretable PROM targets for operative Crohn’s disease. First, these scores may help to conceptualize progress during postoperative visits. This includes within-patient trends, where current scores are compared to a patient’s own baseline, as well as between-patient trends, where current scores are compared to other patients at similar time points in their postoperative course. For example, the current study indicates that most patients transition from below the PASS before the operation to above the PASS after the operation, but that reclassifications continue throughout follow-up though less frequently. Second, these scores may be useful in the preoperative setting for patients who are considering future elective bowel resection. A mismatch between provider and patient expectations has been shown to contribute to postoperative decision regret [32]. Further, the use of PROMs has been associated with greater patient involvement in shared decision-making [33, 34] and perceived quality of decision-making. [33, 35–37] A data-driven tool when discussing plausible trajectories after the intervention may help patients and providers weigh anticipated benefits against risk of complications or lack of improvement. In this study, an association between the preoperative sIBDQ score and score at final follow-up suggests that baseline quality of life may be useful to inform anticipated ceilings in quality of life after Crohn’s-related bowel resection.

There are many limitations to this study. First, the cohort represents Crohn’s disease patients from a single tertiary care center and may not generalize to all practice settings. Thresholds may vary in other populations with different trends in disease characteristics, motivating distinct threshold calculations. For example, stratified analyses according to ostomy status and whether the ostomy was permanent or temporary are an important future direction. Second, unlike many studies that establish clinically meaningful outcome thresholds [11, 38], questionnaires were completed during the routine course of clinical care as opposed to active recruitment with dedicated baseline and follow-up visits. This pragmatic design is most likely to capture patients who regularly seek care and likely misses individuals who pursue alternative opinions at other institutions due to dissatisfaction or limited access to care. Third, although the completion rate of PROMs in the implementation study at our institution exceeded 90% [16], we are unable to identify non-respondents among our current cohort. Fourth, misclassification bias may be present particularly in patients who initially present with findings suggestive of Ulcerative Colitis and later show features of Crohn’s disease such as ileal involvement. Fifth, there is no consensus about ideal methodologies for clinically meaningful PROM thresholds, but our presentation of a range of values with preference for anchor-based strategies is consistent with current recommendations [14, 15]. Sixth, our Crohn’s-related bowel resection analysis did not account for negative postoperative events such as bleeding, anastomotic leaks, or disease recurrence, though these likely contributed to differences in quality of life. In a sensitivity analysis, we stratified final sIBDQ scores by any evidence of postoperative endoscopic disease recurrence and by all-cause 30-day readmissions. Relative to those without the negative postoperative event, the median sIBDQ score at final follow-up was 1 point lower for patients with recurrence and 4 points lower for patients with readmissions (Supplementary Material).

In Crohn’s disease, clinically meaningful targets for quality of life may complement traditional metrics when monitoring progress after operative intervention.

## Supplementary Information

Below is the link to the electronic supplementary material.

Supplementary file1 (DOCX 297 KB)
