# Supplementary material for: Benchmarks after bowel resection in Crohn’s disease: implementing clinically meaningful health-related quality of life targets
Source: Surg Endosc. 2025 Jul 23;39(9):6180–91. doi: 10.1007/s00464-025-11983-z (PMC12408668; doi:10.1007/s00464-025-11983-z)
Supplement: Supplementary file 1 — Supplementary file1 (DOCX 297 KB) [file 464_2025_11983_MOESM1_ESM.docx]

## Supplementary Material

### Inclusion Flowchart


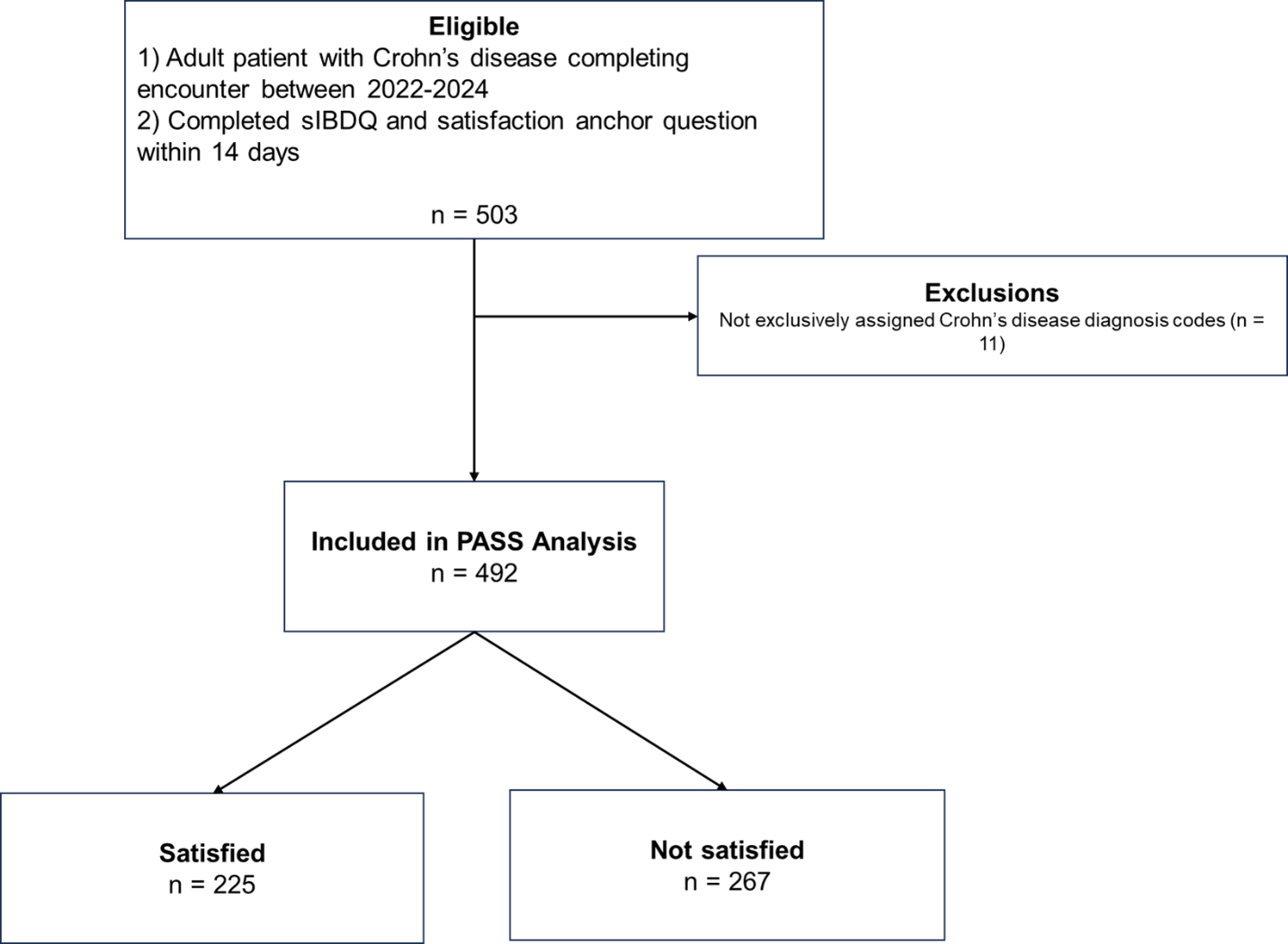


Supplementary Figure 1: Inclusion flowchart for the PASS analysis. PASS: Patient acceptable symptom state. CD: Crohn’s disease. sIBDQ: short inflammatory bowel disease questionnaire.


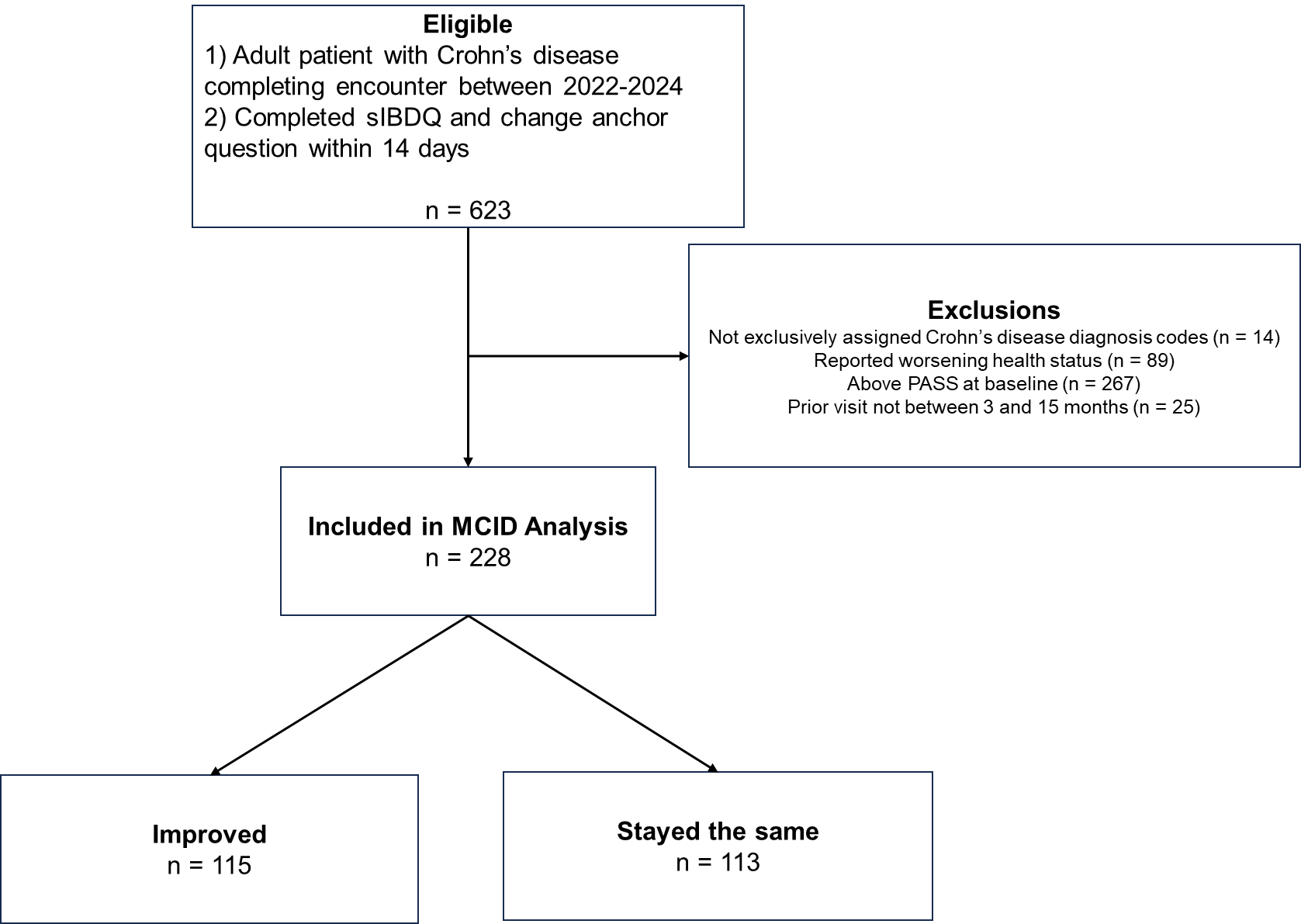


Supplementary Figure 2: Inclusion flowchart for the MCID analysis. MCID: Minimum clinically important difference. CD: Crohn’s disease. sIBDQ: short inflammatory bowel disease questionnaire.


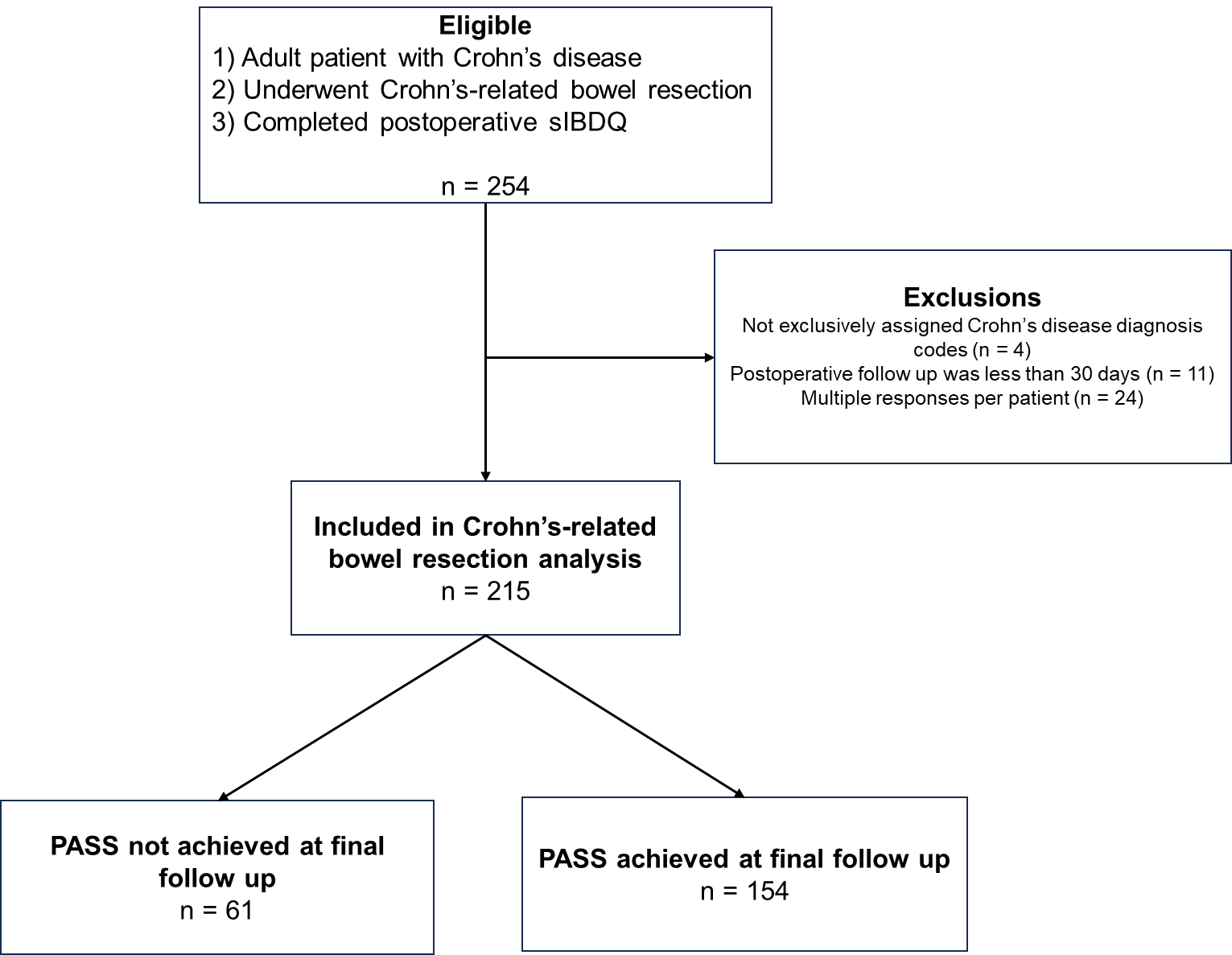


Supplementary Figure 3: Inclusion flowchart for the Crohn’s-related bowel resection analysis. MCID: Minimum clinically important difference. CD: Crohn’s disease. sIBDQ: short inflammatory bowel disease questionnaire.

### Timing of baseline visit for minimum clinically important difference

For the minimum clinically important difference (MCID), we included patients who had a prior visit between 3 and 15 months before their inclusion visit. The three month boundary was selected due to prior work recommending quarterly follow-up intervals for capturing change in health-related quality of life.^1^ From that time point, we included a one year lookback period given the timing of routine follow-up visits at our institution. The distribution of months between visits for the MCID analysis is shown in Supplementary Figure 4.


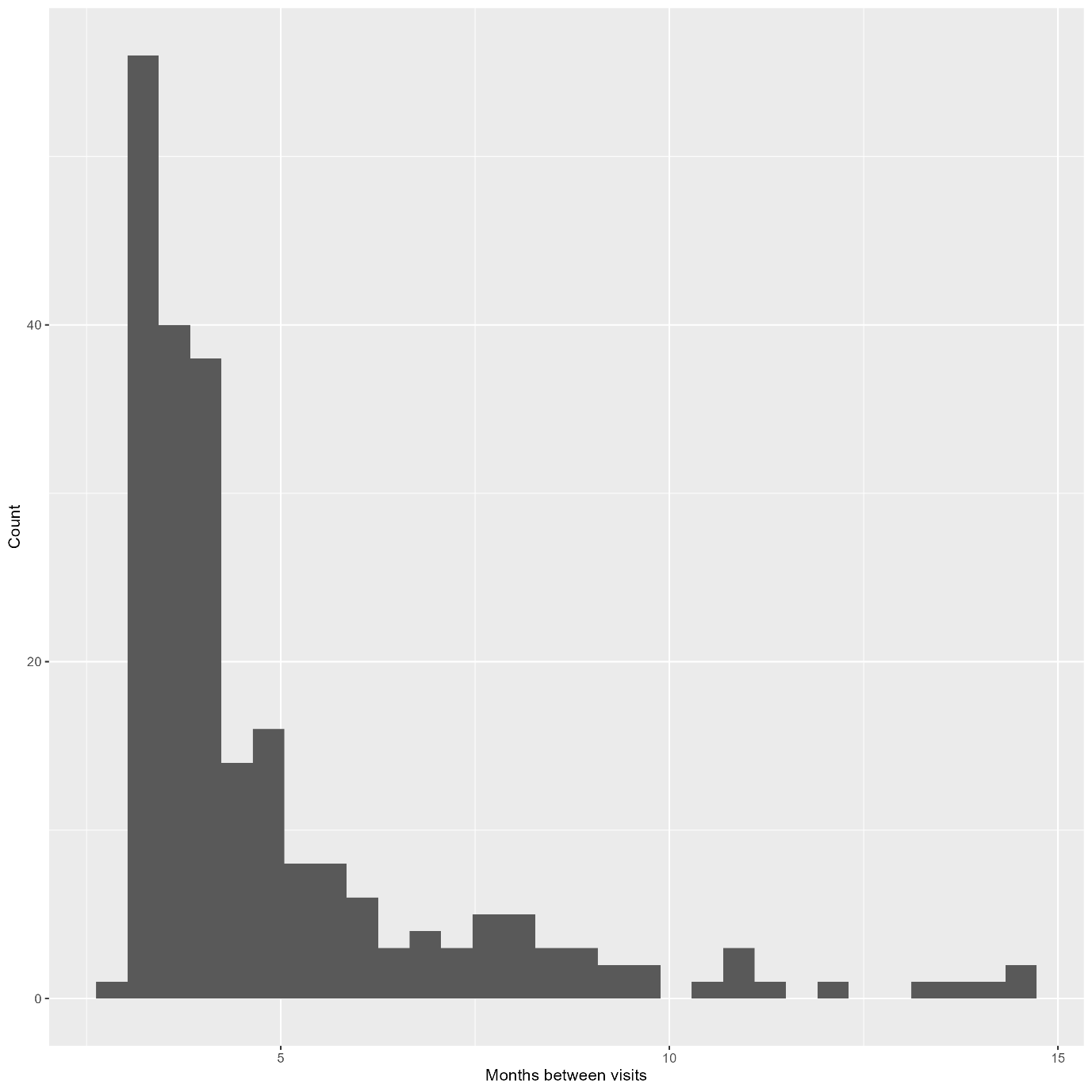


Supplementary Figure 4: Distribution of months between visits for patients included in the MCID analysis. MCID: Minimum Clinically Important Difference

### Crohn’s-related bowel resection analysis

**Procedure codes**

Supplementary Table 1 depicts Current Procedural Terminology, 4^th^ revision (CPT4) codes that were included as part of the Crohn’s-related bowel resection analysis.

| Supplementary Table 1: Crohn’s-related bowel resection procedure codes | |
| --- | --- |
| **Code** | **Description** |
| 44120 | Enterectomy, resection of small intestine; single resection and anastomosis |
| 44121 | Enterectomy, resection of small intestine; each additional resection and anastomosis (List separately in addition to code for primary procedure) |
| 44125 | Enterectomy, resection of small intestine; with enterostomy |
| 44140 | Colectomy, partial; with anastomosis |
| 44141 | Colectomy, partial; with skin level cecostomy or colostomy |
| 44143 | Colectomy, partial; with end colostomy and closure of distal segment (Hartmann type procedure) |
| 44144 | Colectomy, partial; with resection, with colostomy or ileostomy and creation of mucofistula |
| 44147 | Colectomy, partial; abdominal and transanal approach |
| 44150 | Colectomy, total, abdominal, without proctectomy; with ileostomy or ileoproctostomy |
| 44155 | Colectomy, total, abdominal, with proctectomy; with ileostomy |
| 44160 | Colectomy, partial, with removal of terminal ileum with ileocolostomy |
| 44202 | Laparoscopy, surgical; enterectomy, resection of small intestine, single resection and anastomosis |
| 44203 | Laparoscopy, surgical; each additional small intestine resection and anastomosis (List separately in addition to code for primary procedure) |
| 44204 | Laparoscopy, surgical; colectomy, partial, with anastomosis |
| 44205 | Laparoscopy, surgical; colectomy, partial, with removal of terminal ileum with ileocolostomy |
| 44206 | Laparoscopy, surgical; colectomy, partial, with end colostomy and closure of distal segment (Hartmann type procedure) |
| 44210 | Laparoscopy, surgical; colectomy, total, abdominal, without proctectomy, with ileostomy or ileoproctostomy |
| 44212 | Laparoscopy, surgical; colectomy, total, abdominal, with proctectomy, with ileostomy |
| 45110 | Proctectomy; complete, combined abdominoperineal, with colostomy |
| 45111 | Proctectomy; partial resection of rectum, transabdominal approach |
| 45112 | Proctectomy, combined abdominoperineal, pull-through procedure (eg, colo-anal anastomosis) |
| 45114 | Proctectomy, partial, with anastomosis; abdominal and transsacral approach |
| 45116 | Proctectomy, partial, with anastomosis; transsacral approach only (Kraske type) |
| 45123 | Proctectomy, partial, without anastomosis, perineal approach |
| 45395 | Laparoscopy, surgical; proctectomy, complete, combined abdominoperineal, with colostomy |

**Preoperative, Intraoperative, and Postoperative Characteristics**

In the Crohn’s-related bowel resection analysis, Supplementary Table 2 depicts preoperative clinical characteristics when stratified by whether the patient achieved the Patient Acceptable Symptom State (PASS) at any point during postoperative follow-up. Supplementary Table 3 shows operative and postoperative characteristics. Concurrent steroid, immunomodulator, or biologic use referred to active prescriptions within 30 days of the encounter for the following medications: prednisone, prednisolone, methylprednisolone, budesonide, infliximab, adalimumab, golimumab, certolizumab, vedolizumab, ustekinumab, azathioprine, methotrexate, or cyclosporine.

Patients can complete pre-visit questionnaires through the electronic patient portal or while in the waiting room for their clinic visit. In our cohort, questionnaires were completed through the electronic patient portal in 93% of patients. Median (IQR) scores among patients who completed the questionnaire in clinic were 51 (33, 69) compared to 58 (44, 72) among those who completed the questionnaire via the electronic portal.

| Supplementary Table 2: Crohn’s-related bowel resection preoperative characteristics | | |
| --- | --- | --- |
| **Characteristic** | **PASS Not Achieved** N = 61 | **PASS Achieved** N = 154 |
| Age (years) | 41.0 (30.0, 50.0) | 38.5 (28.0, 50.0) |
| Sex |  |  |
| Female | 36 (59%) | 73 (47%) |
| Male | 25 (41%) | 81 (53%) |
| Tobacco use |  |  |
| Current | 22 (37%) | 22 (14%) |
| Prior | 10 (17%) | 37 (24%) |
| Never | 27 (46%) | 95 (62%) |
| Insurance |  |  |
| Private | 31 (51%) | 95 (62%) |
| Government | 24 (39%) | 37 (24%) |
| Other | 6 (9.8%) | 21 (14%) |
| BMI (kg/m2) | 26.2 (22.9, 30.0) | 24.1 (20.5, 27.8) |
| Disease Pattern |  |  |
| Inflammatory | 6 (9.8%) | 27 (18%) |
| Penetrating and/or stricturing | 55 (90%) | 122 (82%) |
| Disease Location |  |  |
| Colonic | 6 (10%) | 26 (18%) |
| Ileal | 22 (37%) | 56 (38%) |
| Ileocolonic | 32 (53%) | 66 (45%) |
| Resection history |  |  |
| No Prior Resection | 27 (44%) | 88 (57%) |
| Prior Resection(s) | 34 (56%) | 66 (43%) |
| Concurrent Steroids | 30 (49%) | 67 (44%) |
| Concurrent Immunomodulators or Biologics | 27 (44%) | 78 (51%) |
| Disease duration (years) | 10.0 (4.0, 18.0) | 11.0 (5.0, 21.0) |
| Extraintestinal manifestations | 3 (4.9%) | 12 (7.8%) |
| Preop sIBDQ score | 38.5 (28.5, 49.0) | 53.0 (42.0, 61.0) |
| Values represent Median (Q1, Q3) or n (%). sIBDQ: Short inflammatory bowel disease questionnaire. BMI: Body Mass Index. PASS: Patient Acceptable Symptom State | | |

| Supplementary Table 3: Crohn’s-related bowel resection operative and postoperative characteristics | | |
| --- | --- | --- |
| **Characteristic** | **PASS Not Achieved** N = 61 | **PASS Achieved** N = 154 |
| Study Resection Approach |  |  |
| Laparoscopic | 38 (62%) | 102 (66%) |
| Open | 23 (38%) | 52 (34%) |
| Follow up duration (months) |  |  |
| 1-12 | 20 (33%) | 52 (34%) |
| 13-24 | 19 (32%) | 36 (24%) |
| More than 24 | 21 (35%) | 64 (42%) |
| 30-day ED visit or readmission | 12 (20%) | 25 (16%) |
| Ostomy present at final follow up | 24 (39%) | 52 (34%) |
| Method of Completing sIBDQ at final follow-up |  |  |
| In Clinic | 8 (13%) | 8 (5.2%) |
| Electronic Portal | 53 (87%) | 146 (95%) |
| Recurrence on postoperative endoscopy | 14 (29%) | 41 (33%) |
| sIBDQ score at final follow up | 42.0 (35.0, 47.0) | 62.0 (57.0, 66.0) |
| Values represent Median (Q1, Q3) or n (%). sIBDQ: Short inflammatory bowel disease questionnaire. BMI: Body Mass Index. PASS: Patient Acceptable Symptom State | | |

**Factors influencing quality of life at final follow-up**

Postoperative trajectories in Crohn’s disease vary widely, and many factors may influence sIBDQ or satisfaction scores. We examined sIBDQ scores at final follow-up from the perspective of postoperative endoscopic recurrence and 30-day all-cause postoperative readmission, which are shown in Supplementary Figure 5. There were 37 (17%) patients with a 30-day readmission, who reported a median (IQR) sIBDQ score of 55 (42, 68) compared to 59 (44, 74) among patients without readmission. There were 174 patients with endoscopic surveillance documented in the postoperative period. Among those patients, 55 (32%) had evidence of any macroscopic disease recurrence. Median (IQR) sIBDQ scores among patients without a recurrence were 58 (41, 75) compared to 57 (43, 70) with a recurrence. Patients with recurrence had a mean (SD) Simple Endoscopic Score of 3.8 (3.2).


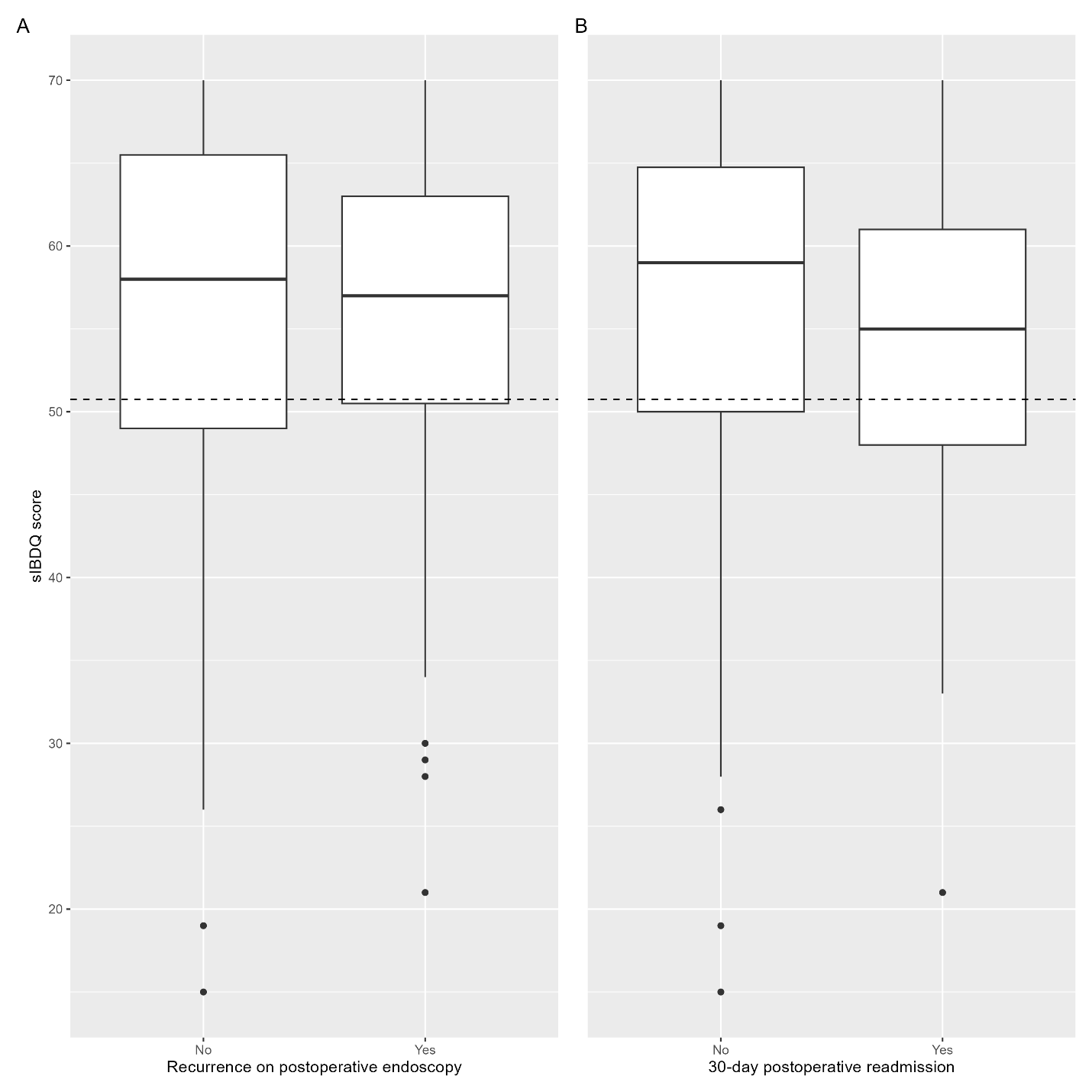


Supplementary Figure 5: sIBDQ scores at final follow up when stratified by a) postoperative endoscopic macroscopic recurrence, and b) 30-day all cause postoperative readmission. Dashed line represents the Patient Acceptable Symptom State score for the sIBDQ, which was 51. sIBDQ: short inflammatory bowel disease questionnaire

**Within-patient variability**

In the Crohn’s-related bowel resection analysis, some patients reported multiple scores within each postoperative time point. The distribution of scores among these patients is shown in Supplementary Figure 6. Across time points, the standard deviation was above the MCID in 16.7% (13-24 month time point), 22.1% (More than 24 month time point), or 33.1% (1-12 month time point).


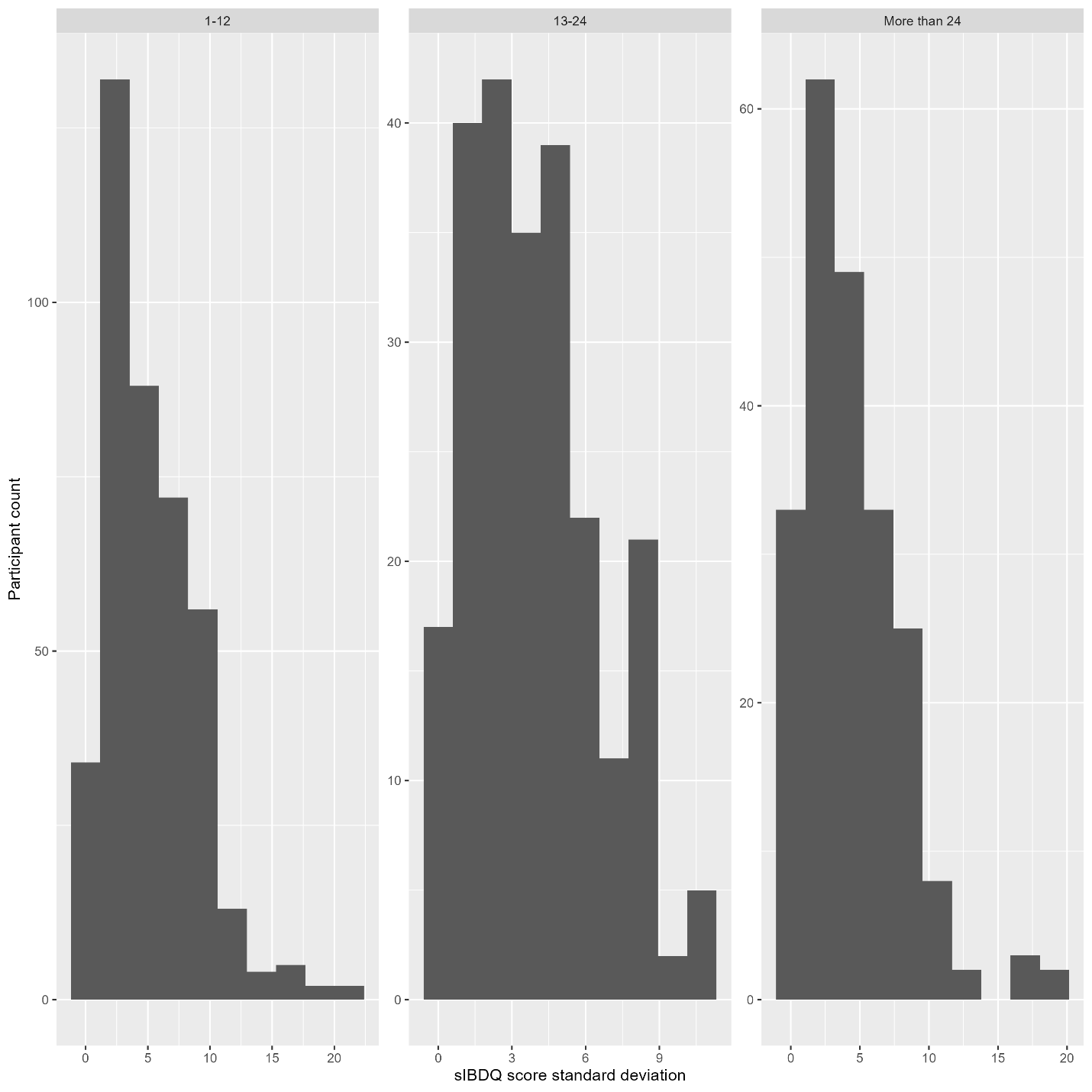


Supplementary Figure 6: Within-patient variability in sIBDQ scores among patients reporting multiple scores for a time point. Time points reflect months after the operation. IBDQ: Short inflammatory bowel disease questionnaire

## References

1. Khor S, Flum DR, Strate LL, et al. Establishing Clinically Significant Patient-reported Outcomes for Diverticular Disease. *Journal of Surgical Research*. 2021;264:20-29. doi:10.1016/j.jss.2021.01.045
